# Magnitude and associated factors of anemia among pregnant women in Dera District: a cross-sectional study in northwest Ethiopia

**DOI:** 10.1186/s13104-017-2690-x

**Published:** 2017-08-01

**Authors:** Terefe Derso, Zelalem Abera, Amare Tariku

**Affiliations:** 1 Department of Human Nutrition, Institute of Public Health, College of Medicine and Health Sciences, University of Gondar, P.O.Box:196, Gondar, Ethiopia; 2 John Snow Integrated Family Health Program, Ethiopia, P.O.Box:1841, Bahirdar, Ethiopia

**Keywords:** Anemia, Pregnant women, Iron supplementation, Northwest Ethiopia

## Abstract

**Background:**

Anemia is associated with adverse health and socio-economic consequences among pregnant women. Particularly, severe anemia increases the risk of maternal mortality by 20%. However, literatures are scarce in the northwest Ethiopia. Therefore, this study aimed to determine the magnitude and associated factors of anemia among pregnant women attending antenatal care in Dera District, South Gondar Zone, northwest Ethiopia.

**Methods:**

A facility-based cross-sectional study was conducted in Dera District health centers from June 27 to September 2, 2015. Capillary blood samples were taken from 348 pregnant women. The raw measured values of hemoglobin were obtained using the portable Hb301 instrument and adjusted for altitude. Besides, nutritional status of the women was assessed by the mid upper arm circumference (MUAC) taken on non-dominant upper limb, mostly of the left hand. Socio-demographic factors, obstetric history, environmental related factors and dietary intake were collected by interviewing the pregnant women. A multivariate logistic regression analysis was employed to identify factors associated with anemia. Adjusted odds ratio (AOR) with corresponding 95% confidence interval (CI) was computed to show the strength of association. In multivariable analysis, a *P* value of <0.05 was used to declare statistical significance.

**Results:**

The overall prevalence of anemia among pregnant women was 30.5% [95% CI 21.0, 40.0]. The result of multivariable analysis revealed that the likelihood of anemia was higher among pregnant women living in rural areas [AOR = 3.03, 95% CI 1.17, 7.82], had no latrine [AOR = 4.75, 95% CI 1.15, 16.60], low monthly income: <Eth. Birr 1200 (US dollar 52.22) [AOR = 2.56, 95% CI 1.17, 5.60], five or above parity [AOR = 4.17; 95% CI 1.62, 10.69], MUAC < 23 cm [AOR = 4.97; 95% CI 2.61, 9.43] and did not prenatal take iron supplementation [AOR = 5.63; 95% 2.21, 14.32].

**Conclusion:**

So far in the district, the magnitude of anemia existed as a moderate public health concern. Thus, improved socio-economic status, latrine and maternal iron supplementation coverage are essential to mitigate the high burden of anemia. In addition, nutritional counseling and education on the consumption of extra meals and iron-rich foods should be intensified.

## Background

Anemia is a serious public health problem among pregnant women in developing countries [1, 2]. About 95.7% of the global burden of prenatal anemia is found in developing countries [3]. Nearly half (46.3%) of the pregnant women in Africa are anemic [4], likewise two-third (62.7%) are suffering from the problem in Ethiopia [2].

The prenatal anemia is associated with adverse health and socio-economic consequences [2]. Impaired physical strength and increased risks of maternal morbidity are common among anemic pregnant women. Particularly, the risk of mortality raises by 20%, in case of severe anemia [5–9]. Also maternal anemia affects the growing fetus and the newborn. The higher odds of fetal anemia, preterm delivery, low birth weight (LBW), intrauterine fetal growth restriction and perinatal mortality are reported among anemic pregnant mothers [7–9].

Because of increased volume of water in the blood, resulting in hemo-dilution and rapid fetal growth pregnant women are highly vulnerable to anemia [10–12]. Cognizant of this condition the World Health Organization (WHO) recommends universal iron-folate supplementation for pregnant women [13].

Based on the previous findings, prenatal anemia is associated with maternal co-existing nutrient deficiencies, obstetric morbidities, socio-demographic and economic characteristics. Accordingly, deficiency of some micronutrients (vitamin C, vitamin B12 and vitamin A) which impairs the absorption and bioavailability of iron [14–16], under-nutrition (MUAC < 23 cm) [17], low meal frequency (<3 times per day) [18] and not taking iron-folate supplementation [19] are related with increased odds of maternal anemia. History of obstetric morbidities, such as intestinal parasitic infestations and recurrent malarial attack are correlated with prenatal anemia [16, 20–22]. Also high risk of anemia is reported among mothers with advanced gestational age [23], high parity [24] and gravidity [25]. Rural residence [21], illiteracy [26], large family size [27] and poor economic status [28] are found the socio-economic determinants of anemia.

Ethiopia recognizes the maternal anemia as a severe public health concern for decades, consequently national nutrition program and the micronutrient deficiency prevention and control strategy has been implemented in the country [29, 30]. Universal iron-folate supplementation is currently given for all pregnant women attending antenatal care (ANC) in Ethiopia [29]. However, only 34% of mothers are found to have at least one antenatal care which makes implementation of universal iron-folate supplementation more challenging. Besides, only 17% of mothers took the supplementation during pregnancy while less than 1% of them took for full schedule (90 days) [31]. Nevertheless, anemia is one of the rampant nutritional problem in Ethiopia, literatures are scarce. Therefore, this study aimed to identify the magnitude and associated factors of anemia among pregnant women attending ANC in Dera District, South Gondar Zone, northwest Ethiopia.

## Methods

### Study setting and design

A facility-based cross-sectional study was conducted in Dera District health centers from June 27 to September 2, 2015. The district is located in South Gondar administrative zone, 602 km from Addis Ababa, the capital of Ethiopia. The altitude of the district ranges from 1500 to 2500 m above sea level. A total of 282,775 populations reside in the district. A total of ten health centers are found in Dera District.

### Study participants and sampling procedure

All pregnant women who attended ANC in Dera District health centers were eligible for the study. To estimate the magnitude of anemia among pregnant women, sample size was calculated using single proportion formula by considering the following assumptions; 16.6% as the prevalence of anemia among pregnant women in Gondar District [32], 95% level of confidence, 5% margin of error, 10% non-response rate and a design effect of 1.5. Thus, the final sample size of 351 was obtained. Regarding the sampling procedure, initially a lottery method was employed to select three health centers out of ten health centers. After estimating the average number of pregnant women attending ANC in the past 2 months using the registration log-book, the proportional allocation was used to calculate the total number of mothers selected from each health center. As a result, 124, 115 and 109 pregnant women were selected using a systematic sampling technique from Ambesame, Aribgebya and Hamusit health centers, respectively.

### Data collection tools and procedure

A structured and pretested questionnaire was used to obtain socio-demographic information, obstetric history, environmental and dietary intake related characteristics of the study participants. The questionnaire was originally prepared in English and then translated to Amharic to local language and back to English in order to obtain content validity. Three BSc clinical nurses and three laboratory technologists were participated as data collector while three senior public health officers were involved as a supervisor. Two days training was given to data collectors and supervisors regarding the objective of the study, confidentiality of information, and techniques of interview. Pre-test was done among 5% of the total sample in Woreta health center. During pre-test the questionnaire was assessed for its clarity, wording and the optimal time for completing the interview. Modifications were done based on the result. The investigators and the supervisors made regular supervision.

### Measurement of hemoglobin concentration and mid upper arm circumference (MUAC)

Capillary blood samples were taken. The raw measured values of hemoglobin were obtained using the portable Hb301 instrument and adjusted for altitude [12, 31, 33]. The outcome variable, anemia was defined as proportion of pregnant women whose blood hemoglobin concentration of less than 11 g/dl. Also anemia was classified into three categories as mild, moderate and severe anemia when the mother’s hemoglobin level was 10–10.9, 7–9.9 g/dl and less than 7 g/dl, respectively [34]. In addition, the mid upper arm circumference (MUAC) was measured on the non-dominant hand, mostly of the left hand. The result was interpreted according to the WHO and Food and Nutrition Technical Assistance Project (FANTA) recommendation, in which a mother was defined as undernourished if her MUAC was less than 23 cm, whereas she was well nourished if the MUAC was greater than or equal to 23 cm [35, 36].

### Data analysis

Data were checked, coded and entered into Epi-info software version 3.5.3 and exported to Statistical Package for Social Science (SPSS) version 20 for analysis. The bivariate and multivariate analysis were done. All variables with a P value of less than 0.2 in bivariate analysis were entered to multivariate analysis to control the possible effect of confounders. Multi-co-linearity was checked for household income and latrine availability, and the result showed that the two independent variables had no significant co-linearity. Both crude odds ratio (COR) and adjusted odds ratio (AOR) with a 95% confidence interval (CI) were estimated to show the strength of association. In multivariate analysis, variables with a P value of ≤0.05 were considered as statistical significant.

## Results

### Socio-demographic and economic characteristics

A total of 348 pregnant women were included in the study with the response rate of 99.1%. The mean (±standard deviation, SD) age of the pregnant women was 26.31 (±5.93) years. Almost all (98.9%) of the pregnant women were married. Nearly half (48.9%) of the pregnant women did not wear shoe. Besides, more than three-fourth (79, 76.7 and 76.4%, respectively) of the pregnant women were farmer, lives in rural areas and had no formal education (Table 1).

**Table 1:** Socio-demographic and economic characteristics of pregnant women attending antenatal care in Dera District health facilities, Ethiopia, 2015

Variables	Frequency (n = 348)	Percent
Age in years		
15–19	37	10.6
20–29	200	57.5
30–39	104	29.9
≥40	7	2
Mean (±SD) age	26.31 (± 5.93)	
Residence		
Rural	267	76.7
Urban	81	23.3
Occupation		
Farmer	275	79
Merchant	40	11.5
Others^a^	33	9.5
Religion		
Christian orthodox	345	98.6
Muslim	5	1.4
Marital status		
Currently married	344	98.9
Currently unmarried^b^	4	1.1
Educational status		
No formal education	266	76.4
Primary school	50	14.4
Secondary school	22	6.3
Above secondary school	10	2.9
Household monthly income		
≤Eth. Birr 500 (US dollar 21.78)	57	16.4
Eth. Birr 501–850 (US dollar 21.8–36.99)	55	15.8
Eth. Birr 851–1200 (US dollar 37.03–52.22)	139	39.9
>Eth. Birr 1200 (US dollar 52.22)	97	27.9
Source of drinking water		
Tap water	140	40.2
Protected spring water	103	29.6
Unprotected spring water	11	3.2
River water	94	27
Wearing of shoes consistently		
Yes	178	51.1
No	170	48.9

*ETB* Ethiopian Birr

^a^Civil servant and housewife

^b^Divorced

### Obstetric and other medical related characteristics

About one-fourth (25.9%) of the participants had four and above parities. Majority (46.3%) of the pregnant women were found in the second trimester (gestational age between 12 and 28 weeks) of pregnancy. Besides, 73% of the pregnant women had history of multi-gravidity. Majority, 325 (93.4%) of the study participants reported that they utilized mosquito bed net (Table 2).

**Table 2:** Obstetric and medical history of pregnant women attending antenatal care in Dera District health facilities, Ethiopia, 2015

Variables	Frequency	Percent
Parity		
No	88	25.3
One	62	17.8
2–3	108	31
4 and above	90	25.9
Space between pregnancy		
<2 years	9	2.6
≥2 years	251	72.1
No child	88	25.3
Gestational age in weeks		
<12	53	15.2
12–28	161	46.3
>28	134	38.5
Number of gravidity		
Primigravida	94	27
Multigravida	254	73
History if vaginal bleeding		
Yes	21	6
No	327	94
History of infection with intestinal parasite		
Yes	78	22.4
No	270	77.6
History of malaria infection		
Yes	82	23.6
No	266	76.4
Current utilization of ITN		
Yes	325	93.4
No	23	6.6

*ITN* insecticide treated net

### Nutrition related characteristics

More than one-third (35.5%) of the pregnant women were undernourished (MUAC < 23 cm). Besides, only 18.1% of pregnant women had a meal frequency of four and above in the previous 24-h preceding the date of survey. Two-third (67%) of pregnant women did not consume animal products, such as red meat, organ meat and egg at least once per week (Table 3).

**Table 3:** Nutrition related characteristics of pregnant women attending antenatal care in Dera District health facilities, Ethiopia, 2015

Variables	Frequency	Percent
MUAC		
Undernourished (MUAC < 23 cm)	125	35.8
Normal (MUAC ≥ 23 cm)	223	64.1
Meal frequency in the previous 24 h		
Twice	86	24.7
Three times	199	57.2
Four and above times	63	18.1
History of iron supplementation for the current pregnancy		
Yes	303	87.1
No	45	12.9
Eating vegetables food groups at least once per week		
Yes	156	44.8
No	192	55.2
Eating animal source food at least once per week		
Yes	115	67
No	233	33

### Prevalence of anemia

**Fig. 1:**
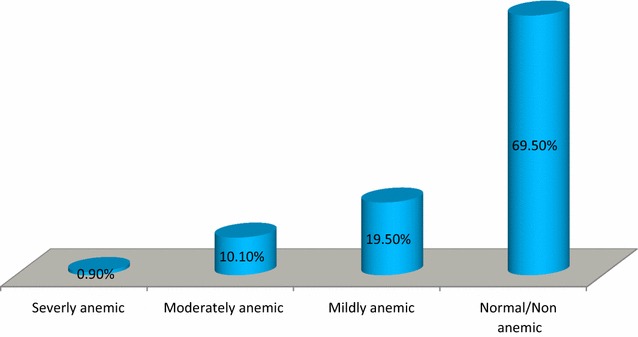
Distribution and severity of anemia among pregnant women attending antenatal care in Dera District health facilities, northwest Ethiopia, 2015

The overall prevalence of anemia among pregnant women was 30.5% [95% CI 21.0, 40.0], of which, one out of ten (10.1%) of the women were moderately anemic (Fig. 1). The mean hemoglobin level (±SD) was 12.07 (±2.24) g/dl.

### Factors associated with anemia

In bivariate analysis residence, parity, household monthly income, availability of latrine, frequency of meal per day, iron supplementation, history of intestine parasite infestation, eating animal source of food at least once per week, history of malaria infection, and nutritional status were found with a P value of less than 0.2. On the other hand, the result of multivariate analysis showed that residence, parity, latrine availability, household monthly income, iron supplementation and nutritional status were significantly and independently associated with anemia. Consequently, the odds of anemia were higher among pregnant women living in rural areas [AOR = 3.03, 95% CI 1.17, 7.82] and in households without latrine [AOR = 4.75, 95% CI 1.15, 16.60]. Increased odds of anemia were observed among pregnant women with MUAC < 23 cm [AOR = 4.97; 95% CI 2.61, 9.43] and who did not take iron supplementation [AOR = 5.63; 95% 2.21, 14.32]. Besides, the likelihoods of anemia was higher among pregnant women who were in the household with an income less than Eth. Birr 1200 (US dollar 52.22) [AOR = 2.56, CI (1.17, 5.60)] and five or more parity [AOR = 4.17; 95% CI 1.62, 10.69] (Table 4).

**Table 4:** Factors associated with anemia among pregnant women attending antenatal care in Dera District health facilities, Ethiopia, 2015

Variables	Anemia		Crude odds ratio (95% CI)	Adjusted odds ratio (95% CI)
	Yes (#)	No (#)		
Residence				
Rural	78	189	4.36 (1.96, 8.08)	*3.03* (*1.17, 7.82*)*
Urban	7	74	1.00	1.00
Parity				
<2 children	24	126	1.00	1.00
2–4 children	61	102	3.14 (1.83, 5.39)	1.74 (0.68, 4.49)
>/=5 children	21	14	7.88 (3.52, 17.61)	*4.20* (*1.14, 15.52*)*
Household monthly income				
<Eth. Birr 2000	91	150	3.72 (2.03, 6.81)	*2.56* (*1.17, 5.60*)*
≥Eth. Birr 2000	15	92	1.00	1.00
Availability of latrine				
Yes	94	238	1.00	1.00
No	12	4	7.60 (2.39, 24.15)	*4.75* (*1.15, 19.60*)*
Gestational age in weeks				
<12	17	36	1.00	
12–28	54	107	1.07 (0.55, 2.17)	
>28	35	99	0.75 (0.45, 1.77)	
Meal frequency in the previous 24 h				
2	41	45	3.19 (1.54, 6.61)	
3	51	148	1.21 (0.62, 2.37)	
4 and above	14	49	1.00	
Iron supplementation in the current pregnancy				
Yes	75	228	1.00	1.00
No	31	14	6.73 (3.40, 13.32)	*5.63* (*2.21, 14.32*)*
Nutritional status				
Normal/MUAC ≥ 23 cm	38	185	1	1.00
Malnourished/MUAC < 23 cm	68	57	5.80 (3.54, 9.53)	*4.97* (*2.61, 9.43*)*

* P value of <0.05

## Discussion

This study revealed that, the overall magnitude of anemia among pregnant women was 30.5%. The result was similar with the former findings in Ethiopia (21.6–36.6%) [17, 21, 31, 37] and northern Nigeria (30%) [38]. However, this prevalence was higher than other reports in Ethiopia, such as Gondar District (16.6%) [32], Awaassa District (15%) [39] and Mekelle District (19.7%) [18] and Iran (13.6%) [40]. The observed discrepancy might be due to rural residence of women (76.7%) in the current study area compared to the latter study settings. Pregnant women living in the rural areas were more likely to be affected with community belief towards poor feeding practices, and had lower access to nutrition education and counseling [31]. Generally, unequal allocation of resources causes to inequalities in women health outcomes between rural and urban settlements. This implies that maternal undernutrition can decline by improving the quality of obstetric care during pregnancy [41].

The result of multivariable analysis also re-affirmed that, the likelihood of being anemic was 3.03 times higher among pregnant women living in rural areas compared to urban residents. Similar results were reported by other local studies [17, 21]. Obviously, pregnant women residing in the rural areas have lack of information about extra nutrient intake during pregnancy and poor access to health care facilities which in-turn increases their vulnerability for anemia [31, 42].

The uncommon finding of this study illustrated a statistical significant association between maternal anemia and unavailability of latrine in the household. Open defecation especially, in high population density areas aggravates the contamination of the household environment [43, 44] and contribute to the risk of developing different intestinal parasitic diseases [45]. The intestinal parasitic infections are the common causes of malabsorption of nutrients, loss of appetite and increased blood loss thereby decreasing the hemoglobin concentration [46–48]. In Africa two-third of pregnant women are at risk of hookworm related anemia [17, 49]. Therefore, improved hygiene and sanitation is a key to reduce risk of anemia, which mainly operates through reducing risk of recurrent intestinal parasitic infestations [41].

The odds of anemia were higher among pregnant women who did not take iron supplementation as compared to their counterparts. The result was supported by what was reported in Vietnam [18]. Though iron requirement increases during pregnancy, poor dietary intake of iron rich food is observed in this study and the 2011 Ethiopia Demographic and Health Survey report [31]. Even the cereal based monotonous dietary habit of the pregnant mothers and other population segments in developing countries including Ethiopia may not help them to ensure their extra requirement for iron [12]. Thus, supplementation of micronutrients, for instance iron and folic acid during pregnancy is not only used to prevent anemia, but it could also help to break the intergenerational cycle of low birth weight, stunting and future child and adult undernutrition [41].

The higher likelihood of anemia was noted among undernourished (MUAC < 23 cm) pregnant women compared to the well-nourished pregnant women. This finding was consistent with the finding from Eastern Ethiopia [50]. This might be related to the negative effect of protein and other macronutrient deficiencies in impairing the bioavailability and storage of iron and other hematopoietic nutrients (folic acid and vitamin B12). As a result, most of the micronutrient deficiencies occurred with protein energy malnutrition, considering this fact the WHO and other local nutritional management guidelines recommend micronutrient supplementation as a routine intervention [51].

This study detected that, mothers with five and above history of parity were found with increased odds of anemia compared to those who had parity of less than two. The parallel result was indicated by the study in Mekelle District [18]. This can be explained by women having high parity is commonly observed with increased susceptibility to hemorrhage and maternal nutritional depletion syndrome [24]. In a healthy pregnancy, hormonal changes lead to an increase in plasma volume which causes reduction in hemoglobin level but does not drop below a certain level (e.g. 11.0 g/dl) [52]. Compared to the non-pregnant state, every pregnancy carries an increased risk of hemorrhage before, during, and after delivery and higher parity aggravates the risk of hemorrhage [24]. On the other hand, a woman with high parity has a large number of children [14], which implies high rate of sharing of available food and other family resources impairing the per capita food intake of pregnant women.

Finally, the odds of anemia were higher among women with household monthly income less than Eth. Birr 1200 compared to those from a household with monthly income of greater than Eth. Birr 1200. The similar findings were reported elsewhere [28, 32]. It was evident that low household monthly income affects the household food purchasing power in kind and amount resulting in household food insecurity [41]. Consequently, people living in the poor households are found with impaired dietary intake and high risk of nutritional deficiencies [32].

This study showed the burden of anemia among the most vulnerable population groups, pregnant mothers, in Dera District where there is scarcity of literature. However, the study has some limitations, as an illustration the cross sectional nature of the study limits measuring the cause and effect relationship between the outcome and the potential determinants. There might be a recall and social desirability bias while measuring dietary information. Also, the serum iron, transferring and ferritin values were not measured and differences of geographic location and seasonality effect were not assessed. The study did not reveal the burdens of soil transmitted helminthes. Lastly, some of the confidence intervals are wide because of small number of cases in some two-by-two cells of variables.

## Conclusions

Nearly one-third (30.5%) of pregnant mothers were anemic in Dera District, indicating anemia as a moderate public health concern. Thus, improving socio-economic status, latrine and maternal iron supplementation coverage are essential to mitigate the burden of anemia. In addition to nutritional counseling and education on the consumption of extra meals and iron-rich foods should be intensified.

## Abbreviations

ANC: antenatal care
FANTA: Food and Nutrition Technical Assistance Project
MUAC: mid upper arm circumference
WHO: World Health Organization
AOR: adjusted odds ratio
COR: crude odds ratio
CI: confidence interval
SD: standard deviation
